# In your eyes: vision of the body alters touch perception in women with eating disorder symptoms

**DOI:** 10.1007/s00426-021-01478-6

**Published:** 2021-04-21

**Authors:** Sofia Sacchetti, Valentina Cazzato, Francis McGlone, Laura Mirams

**Affiliations:** School of Psychology, Liverpool John Moores University, Room 3.13, Tom Reilly Building, Byrom Street, Liverpool, L3 3AF UK

## Abstract

We investigated the effects of non-informative vision of the body on exteroceptive multisensory integration and touch perception in participants presenting with different levels of eating disorder (ED) symptoms. The study employed a sample of women reporting low (low ED; *n* = 31) vs high (high ED; *n* = 34) levels of subclinical ED symptoms who undertook the Somatic Signal Detection task (SSDT). During the SSDT, participants are required to detect near-threshold tactile stimulation at their fingertip with and without a simultaneous light flash next to the stimulated fingertip. Previous research has found that participants have a tendency to erroneously report touch sensations in the absence of the stimulation, and especially when the light flash is presented. In this study, participants completed the SSDT under two conditions: while their hand was visible (non-informative vision), and while their hand was hidden from sight (no vision). Non-informative vision of the hand was found to have a different effect on SSDT performances according to participants’ levels of ED symptoms. High ED participants were better able to correctly detect the touch during the SSDT when their hand was visible. Conversely, for low ED participants, vision of the body was linked to a greater effect of the light in inducing false reports of touch. We suggest that in those with high ED symptoms, vision of the body may exacerbate a predisposition to focusing on external rather than internal bodily information.

## Introduction

The way we perceive bodily sensations depends on a number of environmental and contextual variables that can enhance or reduce perceptive acuity and alter somatosensation (Longo & Sadibolova, 2013). One of the variables that has been found to influence somatosensory perception is the vision of the body. Some studies have shown that directing visual attention towards a body part enhances tactile perception at that stimulated body part, both when the visual information provides useful information about the tactile stimulation (Halligan, Hunt, Marshall & Wade, 1996; Làdavas, Pellegrino, Farnè & Zeloni, 1998, Làdavas, Farnè, Zeloni & di Pellegrino, 2000) and when it does not (Serino, Farnè, Rinaldesi, Haggard & Làdavas, 2007), that is, when the visual input is non-informative.

Indeed, non-informative vision of the body was found to enhance tactile perception in grating orientation tasks by decreasing discrimination thresholds and increasing discrimination accuracy (Taylor-Clarke, Kennett & Haggard, 2004; Cardini, Longo & Haggard, 2011). Alongside, non-informative vision of the body was also found to enhance tactile spatial acuity in terms of reduced two-point discrimination, enhanced amplitude discrimination of above-threshold stimuli and reduced tactile detection thresholds (Tipper et al., 1998, 2001; Kennett, Taylor-Clarke & Haggard, 2001; Serino, Padiglioni, Haggard & Làdavas, 2009; Keizer, Smeets, Dijkerman, van Elburg & Postma, 2012; Harris, Arabzadeh, Moore & Clifford, 2007). In these cases, vision of the body has been thought to improve tactile perception by sharpening tactile receptive fields in the primary cortical somatosensory map (Haggard, Christakou, & Serino, 2007).

Moreover, the vision of the body has been found to facilitate not only the perception of exteroceptive tactile stimuli but also the perception of internal body signals (interoception). Ainley, Tajadura‐Jiménez, Fotopoulou and Tsakiris (2012) and Ainley and Tsakiris (2013) demonstrated that non-informative vision of one’s own face can enhance the detection of heartbeat sensations (namely, interoceptive accuracy). Participants were asked to perform a heartbeat perception task (HPT; Schandry, 1981) while watching a photograph of their face or their reflection in a mirror as compared to a blank screen. Results showed that participants were more accurate in perceiving their heartbeat whilst looking at their face, although the face was a body part unrelated to the perceptual task, and vision of the self was not providing informative data for the completion of the task per se. In contrast, Serino et al. (2007) showed that the vision of a rubber foot does not enhance tactile acuity on the hand. However, it should be noted that interoception and exteroception are two distinct ways to experience the body that correspond to different processes and therefore they cannot always be compared.

Other research has shown, however, that vision of the body can either enhance or diminish perceptive acuity depending on the body part targeted (Tipper et al., 1998: Serino et al., 2007), the type of task used (Longo & Sadibolova, 2013; Press, Taylor-Clarke, Kennett & Haggard, 2004), and participants’ characteristics (Costantini, 2014; Eshkevari et al., 2012). In this respect, Harris et al. (2007) proposed that non-informative vision of the body does not simply enhance somatosensory processing, but rather it induces adaptive changes in tactile sensitivity within a bimodal sensory system. According to this reasoning, after adaptation of tactile receptive fields subsequent to the vision of the body, detection of near-threshold stimuli is impaired, while the discrimination of stimuli is enhanced.

According to the previous paragraph, Tipper et al. (1998), for example, suggested that familiarity of the stimulated body part can modulate the effects of vision on somatosensory processes. In their study, non-informative vision of a familiar body site (such as the face) was found to facilitate the detection of supra-threshold tactile pulses; in contrast, the vision of a less familiar body part (such as the back of the neck) was found to have little impact (neither facilitatory nor inhibitory) on supra-threshold tactile detection.

Moreover, it has been shown that the vision of the body can either enhance or diminish tactile acuity depending on the type of task used. Longo and Sadibolova (2013), for example, found that vision can actively distort touch perception, rather than increase accuracy when participants are asked to estimate the size of a tactile stimulus. In their study, vision of the stimulated body part significantly reduced the perceived size of a tactile stimulus, as compared to vision of an object or of a non-stimulated body part.

Alongside, Press et al. (2004) showed that the complexity of the task can play a role in determining the effects of vision on tactile perception. In their study, non-informative vision only enhanced tactile perception when the task was both difficult and involving a spatial component (making speeded responses in an at-threshold two-point discrimination task). Performance on an easier non-spatial discrimination task (detecting a brief gap in a 250 ms above-threshold vibration) was actually worse when participants viewed the targeted body part as compared to a neutral object.

Accordingly, Mirams, Poliakoff, Brown and Lloyd (2010) found that non-informative vision of the hand increased errors on a non-spatial touch detection task—the Somatic Signal Detection Task (SSDT, Lloyd, Mason, Brown & Poliakoff, 2008). The SSDT involves detecting near-threshold vibrations delivered on the fingertip, where on 50% of trials there is a simultaneous LED flashing next to the targeted finger. During this task, the presence of the light increases incorrect detection of a vibration when it did not actually occur (Lloyd et al., 2008). Mirams et al. (2010) used the SSDT to assess the effects of a second visual variable, that was a vision and no vision of the stimulated hand, on touch perception. Specifically, the study analyzed whether non-informative vision of the hand compared to no vision of the hand would reduce or increase incorrect reports of feeling touch during the task. According to previous research (Press et al., 2004; Harris et al., 2007), vision of the hand would induce a reduced tactile detection, with less reports of touch (i.e., reduce ‘hits’). However, the study showed that during the vision condition, participants were more inclined to make false reports of feeling the touch, and especially on trials when the light flash occurred. The authors suggested that vision of the hand may have raised the focus on interoceptive information to a detrimental degree that led participants to misinterpret internal signals as external touch, which resulted in more errors during the SSDT. This led to the conclusion that non-informative vision of the body may lead to higher somatic interference and ultimately to a less accurate discrimination of touch during the SSDT.

Another variable that may influence the way the vision of the body impacts on perception refers to individual differences and personality characteristics. A fundamental assumption of cognitive approaches to personality and psychopathology is that individuals differ in their response to similar situations because of differences in the way they process incoming stimuli, in terms of both lower-level and higher-level information processing (Öhman, Lundqvist, & Esteves, 2001; Mineka, Rafaeli, & Yovel, 2003; Yovel, Revelle & Mineka, 2005). Accordingly, psychiatric symptoms (i.e., schizophrenic, eating disorder and somatoform symptoms; Ferri et al., 2014; Peled, Ritsner, Hirschmann, Geva & Modai, 2000; Eshkevari et al., 2012) and personality traits (i.e., emphatic abilities and the ability to describe personal experiences; Asai, Mao, Sugimori & Tanno, 2011; Haans, Kaiser, Bouwhuis & IJsselsteijn, 2012) have been related to individual differences in the way participants encode bodily related visual information (Costantini, 2014).

Research in this context has placed a great focus specifically on Eating Disorders (EDs). EDs are a family of psychopathologies characterized by aberrant eating habits and rituals, fear of gaining weight, disturbances in body weight or shape perception, including unawareness of such perceptual disturbances (APA, 2013).

Body perception in EDs has been linked to a greater focus on visual aspects of the body at the expense of other incoming information (Mehling et al., 2009). On a phenomenological level, this heightened focus on visual bodily information manifests itself with excessive concerns and rumination about one’s own physical appearance and body image (Arciero & Guidano, 2000). However, recent evidence suggests this shift of focus to be present also in the context of lower-level sensory processing, with a general over-investment on exteroceptive information coupled with a blunted perception of bodily information coming from within the body (interoceptive deficits). Simply put, ED patients have been deemed to have a preferential reliance on sensory data deriving from the outer world (exteroception) over interoceptive information (Mehling et al., 2009; Arciero & Guidano, 2000). In this regard, informative data come from studies analysing the integration of conflicting visual and internal somatic information about the body, for example using the Rubber Hand Illusion paradigm (RHI; Botvinick & Cohen, 1998). During the RHI, an experimenter strokes a rubber hand placed in front of participants synchronously with their own hand, which is hidden from sight. The synchronous visuo-tactile stimulation induces participants to mislocate the position of their own hand as closer to the rubber hand, and especially ED patients who were found to be more inclined to perceive this illusion (Mussap & Salton, 2006; Eshkevari et al., 2012; Caglar-Nazali et al., 2014). Therefore, these results provide experimental support to the hypothesis that people with EDs may have an increased sensitivity to the visual aspects of body perception (Eshkevari et al., 2012).

According to these data, it could be argued that non-informative vision of the body may be encoded differently in participants presenting with high versus low ED symptoms and therefore have different effects on touch perception. To test this hypothesis, the current study investigated the effects of non–informative vision of the body on tactile perception in participants presenting with low and high ED symptoms. Tactile perception and visuo-tactile integration were analysed using the SSDT paradigm (Lloyd et al., 2008). Replicating the design employed by Mirams et al. (2010), participants underwent the SSDT in two experimental conditions: non-informative vision of the hand and no vision of the hand.

As the bodily self (that is the global, multimodal awareness of one’s own body; Blanke, 2012) in EDs is mostly anchored to exteroceptive coordinates, we hypothesized that vision of the body, by increasing the focus on the bodily self, would lead high symptomatic participants to focus more on exteroceptive tactile information. Therefore, we expected high ED participants to be more sensitive to touch when performing the SSDT during the non-informative vision condition (i.e., make a higher number of ‘hits’ and a lower number of ‘false alarms’). Of relevance here is a previous study from our research group where we found that vision of one’s own face increased sensitivity to touch during the SSDT only in participants presenting with high ED but not low ED symptoms (Sacchetti, Mirams, McGlone & Cazzato, 2020). Conversely, in low ED participants we expected to replicate the results found by Mirams et al. (2010) with a vision of the body increasing false alarms of touch rather than increasing hits. Indeed, as suggested by the authors, in non-symptomatic participants (in the presence of a bodily self (Blanke, 2012) anchored also to interoception), vision of the body is more likely to enhance the focus on interoceptive information, that are not relevant for the task, and are, therefore, erroneously misinterpreted as external touch.

Additionally, the study further investigated whether individual differences in self-reported interoceptive sensibility (EDI-3), body awareness (Body Perception Questionnaire, BPQ), dysmorphic concerns (Dysmorphic Concerns Questionnaire, DCQ) and body dissatisfaction (the body dissatisfaction subscale on the Eating Disorders Inventory-3) could explain participants responses (hits and false alarms) during the SSDT. Specifically, according to the somatic interference hypothesis (by Mirams et al., 2010) that links false alarms to a misperception of interoceptive sensations, we expected participants self-reporting higher difficulties in recognizing inner bodily signals (as measured by the interoceptive sensibility-EDI-3 subscale and the BPQ) to report also higher false alarms during the SSDT. Moreover, we hypothesized that participants showing higher levels of concerns about their physical appearance (as measured by the body dissatisfaction-EDI-3 subscale and the DCQ) would be more strongly influenced by the vision of their body and therefore respond with higher hits in this condition.

## Methods

### Participants

Sixty-nine females were initially recruited from the staff and student population at Liverpool John Moores University (LJMU) and from the general population via advertisements placed around the university campus. The sample size was based on a power analysis using G*Power 3.0.10 (Faul, Erdfelder, Lang & Buchner, 2007), which indicated that overall a minimum sample of *n* = 44 was needed to detect a medium effect (*f *= 0.25) with 95% power, using a mixed design ANOVA (number of groups = 2 × number of measurements = 4) with alpha at 0.05 (two tailed). The sample size was expanded to 69 participants to increase statistical power and to account for potential outliers. Four participants had overall hit rates of over 90% during the SSDT, suggesting that a consistent threshold level had not been achieved for these participants. Therefore, we report the data from 65 participants.

Participants were right-handed (as assessed using the Edinburgh Handedness Inventory; Oldfield, 1971), between 18 and 62 years of age, with no history or present diagnosis of any psychiatric disorder (including EDs), no impairments in tactile perception of the hand, no uncorrectable vision problems and not pregnant. Only females were recruited due to the fact that literature on EDs in males is still scarce, and EDs have been shown to differ significantly in terms of prevalence and phenomenological manifestations between men and women (Stanford & Lemberg, 2012).

Potential participants completed an online version of the Eating Disorder Inventory-3 (EDI-3; Garner, 2004. See the materials and methods section) and were preselected based on their scoring on the ED Risk Composite. The ED Risk Composite is a subscale of the EDI-3 indicating the level of subclinical ED symptoms in healthy subjects, and it is therefore deemed to be an index of the risk for developing an ED. Individuals scoring in the low (Low ED; *n* = 31; Age: *M* = 25.76; SD 10.37) and high (High ED; *n* = 34; Age: *M* = 26.45; SD 7.87) range of the scale were asked to participate in the study. The first (below Q1 = 19) and last quartile (above Q3 = 30) of the normative distribution of the ED Risk Composite in the general population were used as cut-offs for selecting participants (Garner, 2004). The low ED group was therefore formed by participants at a very low risk for developing an ED who scored 19 or below on the ED Risk Composite (*M* = 10.58; SD 5.36), while the high ED group by participants at a higher risk for developing an ED scoring 30 or above on the ED Risk Composite (*M* = 47.59; SD 14.77).

According to the Helsinki declaration of ethical standards, the study was approved by the LJMU’s University Research Ethics Committee, and all participants gave their informed consent to take part. Participation was rewarded with a £5 shopping voucher or ‘participation points’ for course credit for first-year BSc Psychology students.

### Material and measures

**Fig. 1:**
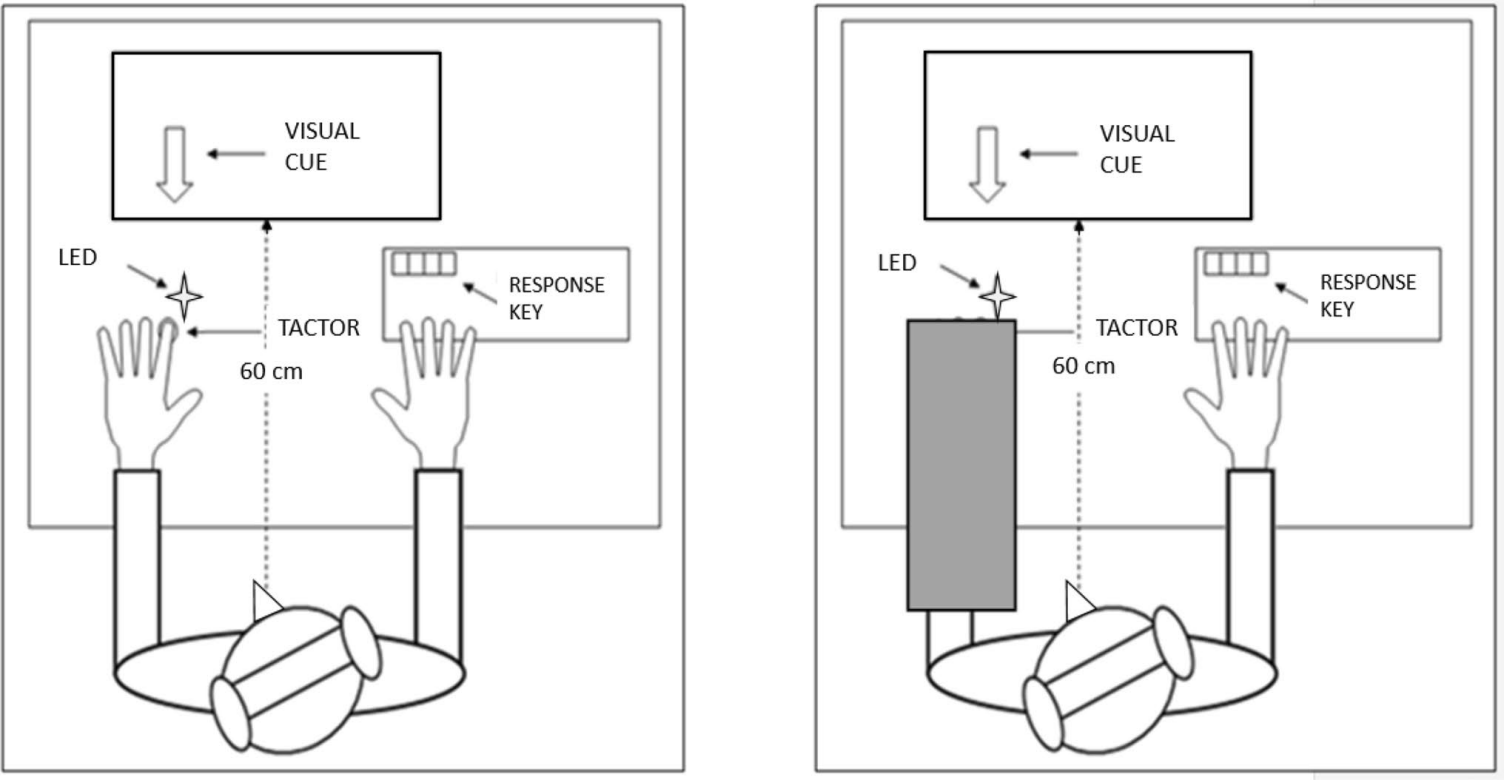
The experimental set-up of the SSDT during the Vision and the No Vision conditions. [Figure created using Microsoft PowerPoint Software (2016)]

*The Somatic Signal Detection Task* (SSDT; Lloyd et al., 2008). Participants sat in a light attenuated room approximately 60 cm in front of a computer monitor. The participant’s left index fingertip was fixed to a tactor delivering vibrations (Z-Voom phones type YVE-01B-03, Yeil Electronics, South Korea) using a double-sided adhesive pad to prevent movements. The tactor was mounted into a polystyrene block, next to a 4 mm light-emitting diode (LED). Tactile pulses (20 ms, 100 Hz vibrations) were produced by a square wave generator connected to the tactor and controlled via E-Prime software (Psychology Software Tools Inc., Pittsburgh, PA, USA). Throughout the experiment, participants listened to white noise via headphones to mask any informative sounds produced by the tactor. Figure 1 illustrates the experimental set-up.

### Self-report questionnaires

#### Eating Disorder Inventory 3 (EDI‐3; Garner, 2004)

The EDI‐3 comprises 91 items organized into 12 primary scales. Three of these scales focus on ED core symptoms: Drive for Thinness, Bulimia and Body Dissatisfaction. The sum of their scores constitutes an index of the risk to develop an ED: the ED Risk Composite. The remaining 9 subscales measure general psychological functioning and other personality traits that have been related to EDs: Low Self-esteem, Personal Alienation, Interpersonal Insecurity, Interpersonal Alienation, Interoceptive Deficit, Emotional Dysregulation, Perfectionism, Ascetism and Maturity Fear. The EDI-3 was administered prior to testing, and the ED Risk Composite was used for selecting eligible participants as explained above. Participants were asked to rate to which extent they considered each item descriptive of themselves on a 6‐point Likert scale ranging from “never” to “always”. The EDI-3 includes items such as “I think that my stomach is too big”, “I am terrified of gaining weight” “I feel inadequate” and “I eat when I’m upset”.

Within the different subscales of the EDI-3, we were particularly interested in results regarding the Interoceptive Deficits subscale, as an index of participants’ self-report ability to correctly recognize and respond to inner bodily states. The subscale was used to investigate whether participants reporting greater difficulties in perceiving interoceptive sensations were also more inclined to report false alarms of touch during the SSDT. The EDI-3 was validated in clinical and non‐clinical samples, and it has been found to have a good internal consistency (*α* = between 0.75 and 0.92 for each subscale), and excellent sensitivity and specificity (Clausen, Rosenvinge, Friborg & Rokkedal, 2011).

#### Dysmorphic Concern Questionnaire (DCQ; Oosthuizen, Lambert, & Castle, 1998)

The DCQ consists of 7 items investigating participants’ concern about their physical appearance. Items cover topics such as the belief of being misshapen or malformed despite others’ opinion; belief in bodily malfunction (e.g. malodour); consultation with cosmetic specialists; spending excessive time worrying about appearance; and spending a lot of time covering up perceived defects in appearance. Participants were asked to rate each item on a Likert scale from a minimum of 0 (“not at all”) to a maximum of 4 (“much more than most people”). Total scores range from 0 to 28 with a critical value of 9 indicating clinical concern (Mancuso, Knoesen & Castle, 2010). The scale was administered to investigate whether individual differences in physical appearance concerns could explain participants’ responses during the SSDT. The DCQ was shown to have a good internal consistency with *α* = 0.80 (Jorgensen et al., 2001).

#### Body Perception Questionnaire-Very Short Form (BPQ-VSF; Porges, 1993)

The BPQ consists of 12 items assessing participants’ awareness about different bodily states associated with changes in the activity of the autonomic nervous system, such as “muscle tension”, “goose bumps”, “stomach and gut pains”, breathing and heart-beat rates. Items are rated on a 5-point Likert scale ranging from 1 (“Never”) to 5 (“Always”). Total scores range between 12 and 60, with higher values reflecting a greater sensitivity and lower values a hyposensitivity to bodily sensations. Together with the Interoceptive Deficit subscale of the EDI-3, the scale was administered to investigate whether individual differences in body awareness were related to participants’ responses during the SSDT. The BPQ has been validated in different samples and has been shown to have a good internal consistency (*α* = between 0.88 and 0.97), and an excellent test–retest reliability (Cabrera et al., 2018).

### Design and procedures

The SSDT protocol consisted of a 2 (Vision/No Vision) × 2 (Light/No Light) × 2 (Touch/No Touch) within subjects design. Each participant underwent all experimental conditions in a repeated measures fashion.

Before beginning the SSDT, participants completed a thresholding procedure to individually calibrate the strength of vibration according to the Parameter Estimation by Sequential Testing (PEST; Taylor & Creelman, 1967) algorithm. A series of pairs of trials were presented. The beginning of each trial was signalled by a white arrow appearing on the left corner of the computer monitor for 250 ms and pointing towards the participant’s left index finger. Each trial had a duration of 1,020 ms. During either the first or the second trial, a 20 ms tactile pulse (Touch) was delivered with a delay of 500 ms on either side. In the other trial, an empty 1020 ms period occurred (No Touch). Participants were then asked to decide whether they had felt a pulse during the first or second trial by pressing the “1” or “2” key on the computer keyboard (a two-alternative forced-choice design). Participants were instructed to keep their hand still throughout the experiment including break and rest periods.

The PEST procedure was set to identify the intensity necessary for participants to detect the vibration in 75% of trials (75% threshold; Mirams, Poliakoff & Lloyd, 2017). The procedure began by presenting the same above threshold vibration to all participants. If participants responded correctly on a series of trials (> 75% correct responses), the programme automatically reduced the strength of the vibration. If they began to respond incorrectly (< 75% correct), the programme automatically increased the vibration strength. A Wald (1947) sequential likelihood-ratio test was used to determine when to change the strength of the vibration. The thresholding procedure took approximately 15 min. If the minimum step size was not reached after 120 trials, the vibration strength was set to the average stimulus strength over the last 50 thresholding trials.

The subsequent SSDT task consisted of two blocks of 80 trials. The tactile pulse was administered in 50% of trials. Simultaneously, the LED flashed in 50% of trials, giving the following four trial types: light only (Light/No Touch); light and touch (Light/Touch); touch only (No Light/Touch); and catch (No Light/No Touch). Each trial type was presented 20 times per block in random order. As for the thresholding procedure, the beginning of each trial was signalled by the appearance of a white arrow cue on the left corner of the monitor for 250 ms. In Touch trials, the tactile stimulus was presented at the threshold level previously established. In No Touch trials, no stimulation was administered. Touch only and catch trials were equivalent to those of the thresholding procedure. In light and touch trials, the LED flashed for 20 ms at the same time as the vibration. In light only trials, the LED flashed for 20 ms alone. At the end of each trial, participants were asked to report whether or not they felt a vibration. They were instructed to press the keyboards buttons ‘1’ for ‘definitely yes’, ‘2’ for ‘maybe yes’, ‘3’ for ‘maybe no’, or ‘4’ for ‘definitely no’. For the purposes of the study, ‘definitely’ and ‘maybe’ responses were combined in a yes/no binary coding.

Each participant completed the SSDT under two conditions: Vision and No Vision of the hand. Each condition consisted of one block of trials. In the Vision condition, participants were able to see the stimulated hand but not the tactile stimulation (which originated from the tactor affixed to their fingertip). During the Vision condition, therefore, non-informative bodily-related visual information were present. In the No Vision condition, the stimulated hand was not visible but hidden by a black cardboard box. The experimenter ensured that the LED was still visible. Participants were instructed to direct their gaze towards the stimulated finger in both conditions. The experimenter remained present throughout each experimental session and ensured that each participant followed this instruction. The order of the Vision and No Vision condition was randomized between participants.

After completing the SSDT, participants were asked to complete the self-report questionnaires. The testing procedure lasted 75 min per participant. Participants were naïve as to the true purpose of the study and were debriefed by the experimenter at the end of the testing session.

## Results

### Data processing

 Responses on the SSDT were classified as hits (reports of feeling the touch on Touch trials), misses (reports of not feeling the touch on Touch trials), false alarms (erroneous reports of feeling the touch on No-Touch trials) or correct rejections (reports of not feeling the touch on No-Touch trials; Mirams et al., 2010). According to the log-linear correction, hit rates (HR) were calculated using the formula [hits + 0.5/(hits + misses + 1)], and false alarm rates (FA) following the formula [false alarms + 0.5/(false alarms + correct rejections + 1)] (Snodgrass and Corwin, 1988). In accordance with the signal detection theory test statistics, participants’ perceptual sensitivity (*d*′) [*z*(hits) − *z*(false alarms)] and tendency to report stimuli as present (response criterion, *c*) [− 0.5 × *z*HR + *z*FA] were calculated using HR and FA (Macmillan and Creelman, 1991). Lower scores on *c* (*c *< 0) indicate a higher tendency to report touch (answer “yes”) across trials.

### Demographics and self-reports analyses

Statistical analyses were performed using SPSS (SPPS Inc., Chicago, IL). All data are reported as Mean (*M*) and Standard Deviation (SD). A significance threshold of *p* < 0.05 was set for all effects, and effect sizes were estimated using partial eta square (*η*^2^) and Cohen’s *d*.

To test for differences in demographics, SSDT threshold levels, and levels of ED psychopathology between the high and low ED groups, a series of t tests were performed with Group as the independent variable and Age, SSDT threshold, Body Mass Index (BMI), scores on each of the EDI-3 subscales, and scores on the DCQ and BPQ as dependent variables. Results of the t tests are presented in Table 1. The two groups were comparable in Age and SSDT threshold levels. There was a significant difference regarding BMI, with the high ED group having a significantly higher BMI than the low ED group. Moreover, the high ED group showed higher scores on most of the subscales of the EDI-3 (Low Self Esteem, Personal Alienation, Interpersonal Insecurity, Emotional Dysregulation, Ascetism and Maturity Fear) indicating a strong link between ED symptoms and other psychological constructs that have been typically related to EDs in our sample. Specifically, the high ED compared to the low ED group reported lower levels of self-esteem, a stronger propensity to feel emotional emptiness and to show reticence in social situations, a tendency toward mood instability and self-denial, and a stronger desire to retreat to the security of childhood. However, differences between the two groups did not reach significance on the Interpersonal Alienation and Perfectionism subscales of the EDI-3, suggesting that the high ED and the low ED groups were comparable in their attitude towards close relationships and in perfectionistic traits. Interestingly, the high ED group also reported a higher difficulty in recognizing and responding to inner body signals as indicated by higher scores on the Interoceptive Deficits subscale of the EDI-3. However, these results were not paired with an equivalent between-groups difference on the BPQ. Conversely, the two groups were found to self-report similar levels of awareness about their bodily states on this scale. The high ED group showed also to have stronger dysmorphic concerns as indicated by higher scores on the DCQ.

**Table 1:** Descriptive statistics for Age, SSDT threshold levels, and questionnaire scores in each group

	Low ED	High ED				
	*M* (SD)	*M* (SD)	*t*	*df*	sig	*d*
Age	26.45 (7.87)	25.76 (10.37)	0.30	63	0.77	0.07
Threshold	− 1190.32 (431.07)	− 1102.29 (585.09)	− 0.69	63	0.49	0.17
BMI	22.48 (4.16)	27.07 (5.68)	− 3.73	63	0.000	0.92
Low Self-esteem	3.80 (4.17)	8.09 (5.02)	− 3.72	63	0.000	0.93
Personal Alienation	3.19 (3.67)	6.26 (4.34)	− 3.06	63	0.003	0.76
Interpers. Insecurity	5.03 (4.73)	8.20 (4.80)	− 2.68	63	0.009	0.66
Interpers. Alienation	4.81 (4.83)	6.03 (3.41)	− 1.19	63	0.24	0.29
Emotional Dysreg.	3.16 (4.24)	6 (5.23)	− 2.39	63	0.02	0.60
Perfectionism	9.16 (5.62)	10.06 (5.03)	− 0.68	63	0.50	0.17
Ascetism	2.80 (2.66)	6.97 (5.25)	− 4.08	63	0.000	1
Maturity Fear	7.23 (4.89)	10.56 (6.13)	− 2.41	63	0.02	0.60
Interoceptive deficit	4.42 (5.23)	8.62 (6.70)	− 2.80	63	0.007	0.70
BPQ	35 (11.58)	33.26 (9.97)	0.65	63	0.52	0.16
DCQ	4.90 (2.96)	8.23 (4.33)	− 3.65	63	0.001	0.90

Low Self-esteem, Personal Alienation, Interpers. Insecurity, Interpers. Alienation, Interoceptive Deficits, Emotional Dysreg. Perfectionism, Ascetism and Maturity Fear are all subscales of the EDI-3

### Main analyses

Descriptive statistics for HR, FA, *d*′ and *c* in each Light and Vision condition of the SSDT, in each group are presented in Table 2. Before performing the analyses, outcomes were tested for normality. HR, *d*′ and *c* were normally distributed, therefore parametric analyses were conducted.

**Table 2:** Descriptive statistics for hit rate, false alarm rate, *d*′ and *c* in each Vision and Light condition and ED group

		HR (%)	FA (%)	FA (%)	*d*′	*c*
		*M* (SD)	Mdn (*IQR*)	*M* (SD)	*M* (SD)	*M* (SD)
*Low ED*						
Vision	Light	60.86 (22.31)	16.67 (0.24)	17.13 (14.46)	1.47 (0.99)	0.39 (0.45)
	No light	59.68 (23.15)	11.90 (0.14)	13.13 (9.6)	1.60 (0.91)	0.25 (0.69)
No vision	Light	65.21 (21.26)	11.90 (0.24)	18.20 (16.87)	1.53 (0.81)	0.50 (0.46)
	No light	56.91 (23.52)	11.90 (0.19)	14.67 (13.45)	1.41 (0.85)	0.52 (0.47)
*High ED*						
Vision	Light	71.20 (15.64)	14.29 (0.19)	19.80 (17.21)	1.65 (0.93)	0.19 (0.36)
	No light	62.76 (22.13)	16.67 (0.20)	17.26 (14.43)	1.54 (1.03)	0.23 (0.53)
No vision	Light	64.05 (18.25)	14.29 (0.25)	21.99 (20.36)	1.35 (0.80)	0.36 (0.44)
	No light	53.22 (20.56)	12.81 (0.19)	14.25 (11)	1.33 (0.78)	0.50 (0.64)

Means (*M*) and the standard deviations (SD) are reported for normally distributed variables (HR, *d*′, *c*), while both M, SD and also medians (Mdn) and inter-quartile ranges (IQR) are shown for the non-normally distributed FA

Specifically, three mixed-design ANOVAs with Group as the between-subject factor, and Light and Vision condition as within-subject factors were performed using HR, *d*′ and *c* as dependent variables. Paired and independent-sample t tests were performed to follow-up significant interactions. Bonferroni correction was used to correct for multiple comparisons. False alarm rates (FA) in each experimental condition showed a significant positive skewness. As FA remained not normally distributed after attempts to transform the data, non-parametric analyses were conducted.

Pearson’s and Spearman’s correlations were used to further investigate the relationships between personality traits and subclinical symptoms (as assessed using self-report questionnaires) and behavioural responses during the SSDT.

#### Hit rate

**Fig. 2:**
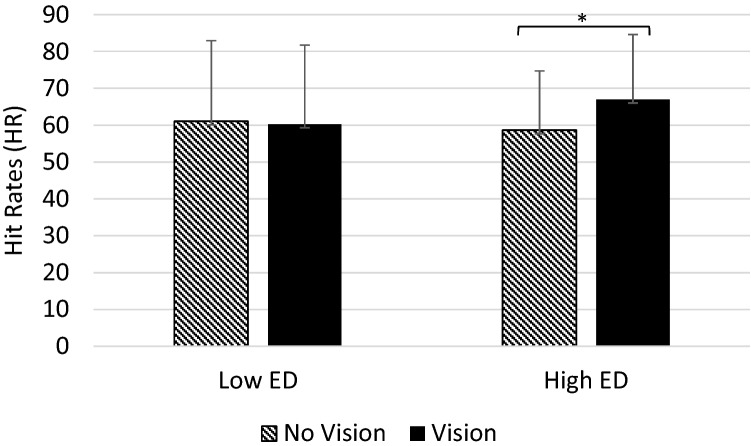
Mean hit rates (HR) during the SSDT in the Vision and No Vision condition in the low and the high ED groups. Error bars show the standard deviation. For the high ED group, there was a significant effect of Vision on hit rates (*p* = 0.003), with higher hit rates in the Vision compared to the No Vision condition. For the low ED group hit rates in the Vision and No Vision condition were comparable (*p* = 0.77). [Figure created using Microsoft Excel Software (2016)]

There was a significant main effect of the Light (*F*(1,63) = 20.70, *p* = 0.000, *η*^2^ = 0.25) indicating that overall subjects had higher HR in light-present (Light; *M *= 60.2, SD 16.17) compared to light-absent trials (No Light; *M *= 58.14, SD 20.15). Alongside, there were a significant main effect of Vision (*F*(1,63) = 4.06, *p* = 0.048, *η*^2^ = 0.06), which was further corroborated by a significant Vision × Group interaction (*F*(1,63) = 5.93, *p* = 0.018, *η*^2^ = 0.09; Fig. 2). Follow-up paired-sample t tests revealed that a significant effect of Vision was present only in the high ED group (*t*(33) = 3.22, *p* = 0.003, *d* = 0.55) but not in the low ED group. High ED participants showed higher HR in trials during which the hand was visible (Vision condition; *M *= 66.98, SD 17.60) compared to trials during which the hand was not visible (No Vision condition; *M *= 58.64, SD 16.03). Conversely, the low ED group seemed not to be affected by the manipulation of the vision of the hand, reporting similar HR in the Vision (*M *= 60.27, SD 21.45) and the No Vision condition (*M *= 61.06, SD 21.88; *t*(30) = − 0.29, *p* = 0.77, *d* = 0.05). Independent-sample t tests showed no between-group differences in HR neither in the Vision nor in the No Vision condition (*t* ≤ 0.51; *p* ≥ 0.17).

There was no significant main effect of Group and no other significant two and three-way interactions (all *Fs* ≤ 3.59; all *ps* ≥ 0.06). However, the Light × Vision interaction was found to approach significance (*F*(1,63) = 3.59, *p* = 0.06, *η*^2^ = 0.05) indicating an additive effect of Light and Vision with higher HR when both visual stimuli were present (Light and Vision; *M *= 66.27, SD 19.66) and lower HR when both visual stimuli were absent (No Light and No Vision; *M *= 54.98, SD 21.92).

Pearson’s r correlations were performed to analyze whether participants’ concerns with their physical appearance (assessed using the body dissatisfaction subscale of the EDI-3 and the DCQ) could explain a greater tendency to focus on visual information, as reflected by higher HR during Light trials of the Vision condition. Results showed a significant positive correlation between body dissatisfaction and HR during Light trials of the Vision condition (*r* = 0.28; *p* = 0.023), so that in the overall sample participants who showed greater dissatisfaction towards their body were also more inclined to report HR in the presence of both visual information: the Vision of the hand and the Light. Results remained significant after performing a Bonferroni correction. However, the DCQ was found not to significantly correlate with HR in Light trials of the Vision condition (*r* ≤ 0.16; *p* ≥ 0.19).

#### False alarms

**Fig. 3:**
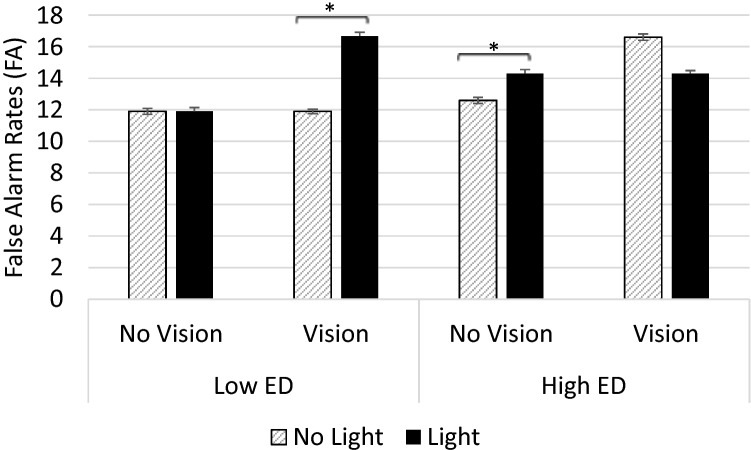
Median false alarms (FA) during the SSDT in the Light and No Light trials of the Vision and No Vision condition in the low and the high ED groups. Error bars show the interquartile range. For the high ED group, there was a significant effect of the Light only in the No Vision condition (*p* = 0.025). For the low ED group, there was a significant effect of the Light only in the Vision condition (*p* = 0.034). Hence, the Light was more likely to induce false alarms when the hand was not visible (No Vision) in high ED participants, and when the hand was visible (Vision) in low ED participants. [Figure created using Microsoft Excel Software (2016)]

A Wilcoxon test showed that FA were significantly affected by the presence of the Light (*z* = − 3.30, *p* = 0.001, *r* = 0.41). Participants were more inclined to misperceive the touch in light-present (Light; Mdn = 31.26) compared to light-absent trials (No Light; Mdn = 25.26). The effect of the Light was present both during the Vision (*z* = − 2.23, *p* = 0.025, *r* = 0.28) and the No Vision conditions (*z* = − 2.66, *p* = 0.008, *r* = 0.33). However, when repeating the analyses separately for the high and for the low ED groups, results showed a significant effect of the Light only in the No Vision condition for the high ED group (Light: Mdn = 14.29; No Light: Mdn = 12.81; *z* = − 2.24, *p* = 0.025, *r* = 0.38) and a significant effect of the Light only in the Vision condition for the low ED group (Light: Mdn = 16.67; No Light: Mdn = 11.90; *z* = − 2.12, *p* = 0.034, *r* = 0.40). Hence, the Light was more likely to induce FA when the hand was not visible in high ED participants, and when the hand was visible in low ED participants (see Fig. 3).

However, and conversely to the results of Mirams et al. (2010), no differences in FA were found between the Vision and the No Vision condition in either Light or No Light trials (*z* ≤ − 0.53; *p* ≥ 0.59). Results remained not significant when the analyses were repeated separately for the low and the high ED group (*z* ≤ − 0.21; *p* ≥ 0.26). Therefore, although visual inspection of the data suggested otherwise, for the low ED group, FA in Light trials were not significantly different in the Vision compared to the No vision condition (*z* ≤ − 21; *p* ≥ 0.83). Moreover, Mann–Whitney tests revealed no overall differences between the two groups (*U* ≤ 471; *p* ≥ 0.39).

Spearman’s *Rho* correlational analyses were run to investigate the relationship between participants’ tendency to focus on inner body signals (assessed using both the BPQ and the Interoceptive Deficits subscale of the EDI-3) and FA during the SSDT. Results showed a positive association between FA and scores on the Interoceptive Deficit (*r* = 0.28; *p* = 0.02) subscales of the EDI-3. Participants who self-reported to have stronger difficulties in recognizing interoceptive signals were also more inclined to report FA during the SSDT across the different experimental conditions. Results remained significant after performing a Bonferroni correction. However, no significant correlations were found between scores on the BPQ and FA (*r *≤ 0.06; *p* ≥ 0.62).

#### Sensitivity (*d*′)

There were no significant main effects of Light, Vision or Group, and no significant two or three-way interactions (all *Fs* ≤ 2.97; all *ps* ≥ 0.09). However, there was a tendency towards a main effect of Vision (*F*(1,63) = 2.97, *p* = 0.09, *η*^2^ = 0.04) due to a higher *d*′ in the Vision compared to the No Vision condition, probably driven by the presence of significantly higher HR in the Vision Vs. the No Vision condition for the high ED group (see above).

#### Response criterion

There was a significant main effect of Vision (*F*(1,63) = 16.17, *p* = 0.000, *η*^2^ = 0.20) with higher *c* in the No Vision condition (*M *= 0.47, SD 0.45) and lower *c* in the Vision condition (*M *= 0.27, SD 21.45), indicating that participants were overall more inclined to report feeling the vibration in the Vision condition compared to the No Vision condition, regardless whether the vibration was administered or not. There were no significant main effects of Light and Group, and no two or three-way interactions were significant (*F* ≤ 2.21; *p* ≥ 0.14).

Overall, significant results in HR and FA were not coupled with significant results in *d*′ or *c*. Therefore, participants’ differences in detecting correctly or not the touch were not better explained by variations in the perceptual sensitivity or in the perceptual bias to report touch regardless of the type of trial.

## Discussion

The aim of this study was to investigate the effects of non-informative vision of the body on exteroceptive multisensory integration and touch perception in participants presenting with different levels of ED symptoms. Based on previous literature that linked EDs with a greater focus on exteroceptive bodily information (Mehling et al., 2009; Arciero & Guidano, 2000; Arciero et al., 2003; Mazzola et al., 2014), we expected high ED participants to be more sensitive to tactile stimuli during the SSDT while viewing their body (in the Vision condition). Conversely, in line with previous results found by Mirams et al. (2010), we expected low ED participants to report a lower sensitivity to touch and higher false alarms in the Vision condition due to a higher level of somatic interference in this condition.

Supporting our expectations, high ED participants were better able to correctly detect the touch during the SSDT when their hand was visible as compared to when their hand was hidden from sight, having a significantly higher HR in the Vision compared to the No Vision condition. Moreover, in high ED participants there was an effect of the Light on FA only in the No vision condition but not in the Vision condition. Therefore, the presence of the light was found to induce false reports of touch only when their hand was hidden from sight. Conversely, when the hand was visible, high ED participants were found to be less affected by the influence of the light, ultimately leading to less FA and a more accurate perception of touch.

These results are in line with arguments that body perception in EDs is characterized by a differential processing of exteroceptive bodily information. Indeed, EDs have been described by the phenomenological psychology as having an “outward dispositional affective style”, which means that ED patients tend to anchor their bodily self to a greater extent to external bodily reference points in the service of visceral and internal somatic information (Arciero & Guidano, 2000; Arciero et al., 2003; Mazzola et al., 2014). In other words, body perception in EDs has been described as showing an over-investment on sensory information deriving from the interactions with the outer world, such as exteroceptive visual and tactile information. Coherently, the vision of the body (as manipulated experimentally) is thought to exacerbate this dispositional perceptive style leading to a greater focus on information, such as touch in the context of the current study. Specifically, we suggest that in participants presenting with ED symptoms, the vision of their body increases attention only towards those dimensions of the bodily self that are already invested by a greater focus, that is exteroception. In turn, this shift of focus determines a greater accuracy in exteroception, and therefore also in detecting tactile stimuli. Interestingly, results of this study are in line with a previous study from our research group (Sacchetti et al., 2020) in which vision of the face, instead of the hand, was found to increase correct detection of touch during the SSDT in high ED participants. Taken together the two studies suggest a consistent effect of vision of the body (across different body parts; i.e., the face and the hand) in enhancing touch detection in EDs.

Conversely, as the bodily self in EDs is not anchored to interoceptive information, the vision of the body may not enhance interoceptive sensibility. Supporting this theory, the high ED group self-reported more confusion and difficulties in recognizing and responding to internal bodily states, as indicated by higher scores in the Interoceptive Deficit scale of the EDI-3.

Furthermore, it should be noted that results in the overall sample showed a positive correlation between HR in Light trials of the Vision condition and the Body Dissatisfaction subscale of the EDI-3. This indicates that participants presenting with a greater dissatisfaction towards their body were also more accurate in perceiving touch when multiple visual information accompanied the stimulation (the presence of the light and the non-informative vision of the hand). These results may suggest that Body Dissatisfaction specifically, among the different subscales of the EDI-3 accounts for the fact that vision of the hand increased HR only in the high ED but not in the low ED group.

For the low ED group, non-informative vision of the body was found not to impact participants’ ability to correctly detect touch. Replicating previous results by Mirams et al. (2010), low ED participants were found to report comparable HR in the Vision and in the No Vision conditions. Moreover, results showed that for the low ED group the presence of the light was more likely to induce false reports of touch only when they performed the task while their hand was visible. Therefore, conversely to the high ED group, for the low ED group, non-informative vision of the hand was found to reduce tactile accuracy by increasing FA. However, conversely to Mirams et al. (2010), and against our expectations, there was no overall difference in FA between the Vision and the No Vision condition. Nevertheless, our results partially support the somatic interference hypothesis, according to which non-informative vision of the body may increase somatic interference arising from internal bodily signals that are mistaken for the external touch.

With this regard, we found a positive correlation between scores on the interoceptive deficit subscale of the EDI-3 and FAs, suggesting that difficulties in recognizing interoceptive information are associated with an increased tendency to erroneously report touch. This is also in line with previous findings that FA during the SSDT are associated with lower levels of interoceptive accuracy as assessed using a Heartbeat Perception Task (HPT; Durlik et al., 2014). This explanation is also consistent with Lloyd et al. (2008) attentional account of touch misperception during the SSDT, according to which attention to the body can increase somatic disturbances by raising awareness of subtle bodily sensations that are confused with external tactile stimuli. A similar process has been used to explain somatoform symptoms, that is physical symptoms experienced in the absence of any apparent physical abnormality (APA, 2013). Different lines of research have linked somatoform symptoms to a heightened and maladaptive awareness of the body that causes an increased salience of benign bodily sensations that are then mistaken for evidence of serious illness (Mehling et al., 2009; Brown et al., 2012). Coherently, previous research on this topic has shown that participants experiencing higher levels of somatoform symptoms are also more inclined to report false sensation of touch during the SSDT (Brown et al., 2010, 2012), possibly due to a hypervigilance towards inner body signals.

Overall, in Mirams et al. (2010), and in the low ED group of the current study, it might be possible that vision of the body amplified and distorted the focus on interoceptive sensations, which were therefore mistakenly confused for exteroceptive signals. This in turn would lead to greater false reports of touch. Interestingly, this effect was specific for the low ED group, and it was not found for the high ED group, where the perception of interoceptive information has been characterized as blunted in EDs (Mehling et al., 2009).

In conclusion, the results of this study indicate that non-informative vision of the body can have different effects on touch perception depending on participants’ level of ED symptoms. Previous research has shown that non-informative vision of the body can either enhance or be detrimental to touch perception depending on the type of task that participants are required to performed, and the familiarity of the body part stimulated (Longo & Sadibolova, 2013; Tipper et al., 1998). However, our results indicate that top-down mechanisms involving participants’ relationship with their body (and specifically body dissatisfaction) can also play a role in determining the effects of non-informative vision of the body on touch perception. Specifically, whereas vision of the body was found to increase the correct detection of touch in participants presenting with high ED symptoms; in participants presenting with low ED symptoms, vision of the body was found to diminish tactile accuracy, by increasing the effect of the Light on false alarms, possibly due to a higher somatic interference of internal body sensations.

Results of this study not only inform current phenomenological models on body perception, but also suggest indications for the clinical field. Indeed, body misperception is still scarcely addressed by different national and international guidelines for the treatment of EDs (Cuzzolaro & Fassino, 2018). It is possible that recovery from EDs could benefit from a partial shift of focus from external to internal bodily information, therefore leading to a rebalance between exteroception and interoception.
